# Supplementary material for: Perceptual awareness of near-threshold tones scales gradually with auditory cortex activity and pupil dilation
Source: iScience. 2024 Jul 17;27(8):110530. doi: 10.1016/j.isci.2024.110530 (PMC11338958; doi:10.1016/j.isci.2024.110530)
Supplement: Document S1. Figures S1–S5, Tables S1, and S2 [file mmc1.pdf]

iScience, Volume *27*

Supplemental information

Perceptual awareness of near-threshold tones scales gradually with auditory cortex activity and pupil dilation

Laura Doll, Andrew R. Dykstra, and Alexander Gutschalk

Supplemental information

Table S1. Results of temporal permutation cluster test,, related to Figure 3, Figure 4, and Figure 6. The time courses were tested in different windows: 100-400ms in the AC, 300-800ms in the RSC/PCC and 500-1000ms in the PDR’. Significant clusters (p<0.05) are marked with an asterisk, dashes indicate, that no cluster was found. In some conditions, two separate clusters were found in the respective time window.

		**AC**			**RSC-PCC**			**PDR‘**		
		t [ms]	*p*		*t [ms]*	*p*		*t [ms]*	*p*	
**Experiment 1:** *one-sided t-tests vs. signal-absent trials*										
Run 1	detected AM	100-230	0.0001	*	331-776	0.0002	*	500-997	0.00002	*
	missed AM	125-169	0.0614		-	0.0911		643-965	0.0030	*
		174-193	0.1069		-	0.0706				
	NT	-	-		-	0.1782		-	-	
Run 2	AM	100-219	0.0001	*	-	-		631-714	0.1075	
		311-400	0.0353	*						
	detected NT	102-400	0.00002	*	395-745	0.0002	*	542-964	0.00002	*
	missed NT	104-400	0.0003	*	-	-		647-906	0.0020	*
Run 3	detected AM	129-192	0.0389	*	335-770	0.00002	*	500-994	0.00002	*
	missed AM	-	-		501-526	0.2158		598-728	0.0113	*
					579-599	0.2323		738-745	0.1941	
					618-682	0.0331	*	918-1000	0.0546	
					692-707	0.2702				
	NT	-	-		-	-		644-660	0.2175	
								672-775	0.0745	
**Experiment 2:** *one-sided t-tests vs. 0*										
Catch	(ratings 1-3)	-	-		-	-		-	-	
Rating	1	210-305	0.0233	*	736-753	0.2499		646-775	0.0499	*
	2	174-400	0.0005	*	374-567	0.0120	*	-	-	
					622-679	0.1177				
					684-710	0.2483				
	3	220-251	0.1116		-	-		-	-	
	4	127-400	0.0014	*	448-468	0.2264		540-790	0.0310	*
					645-677	0.1832				
					770-787	0.2387				
	5	110-400	0.0003	*	406-468	0.1214	*	500-874	0.0002	*
					486-770	0.0019				
	6	100-400	0.0001	*	435-737	0.0004	*	500-909	0.0003	*

Table S2. **Results of temporal permutation cluster test for Figure S2**, related to Figure 3. All time courses were tested against 0 in different windows: 100-400ms in the AC, 300-800ms in the RSC/PCC and 500-1000ms in the PDR’. Significant clusters (p<0.05) are marked with an asterisk, dashes indicate, that no cluster was found. In some conditions, separate clusters were found in the respective time window.

		**AC**		**RSC-PCC**		**PDR‘**	
		t [ms]	*p*	*t [ms]*	*p*	*t [ms]*	*p*
**Group A: NT perceived in run 3**							
Run 1	NT	-	-	598-692	0.0625	-	-
Run 2	detected NT	106-400	0.0156 *	421-702	0.0156 *	536-991	0.0156 *
	undetected NT	129-400	0.0156 *	-	-	632-1000	0.0156 *
Run 3	NT	-	-	447-488	0.1719	673-814	0.0859
				565-749	0.0313 *		
**Group B: NT not perceived in run 3**							
Run 1	NT	-	-	-	-	519-530	0.2266
						547-593	0.1406
						612-629	0.1875
Run 2	detected NT	178-380	0.0391 *	532-679	0.0234 *	511-521	0.2031
				768-786	0.2344	553-917	0.0156 *
	undetected NT	164-238	0.0703	-	-	854-868	0.2031
		359-400	0.1016				
Run 3	NT	-	-	-	-	-	-

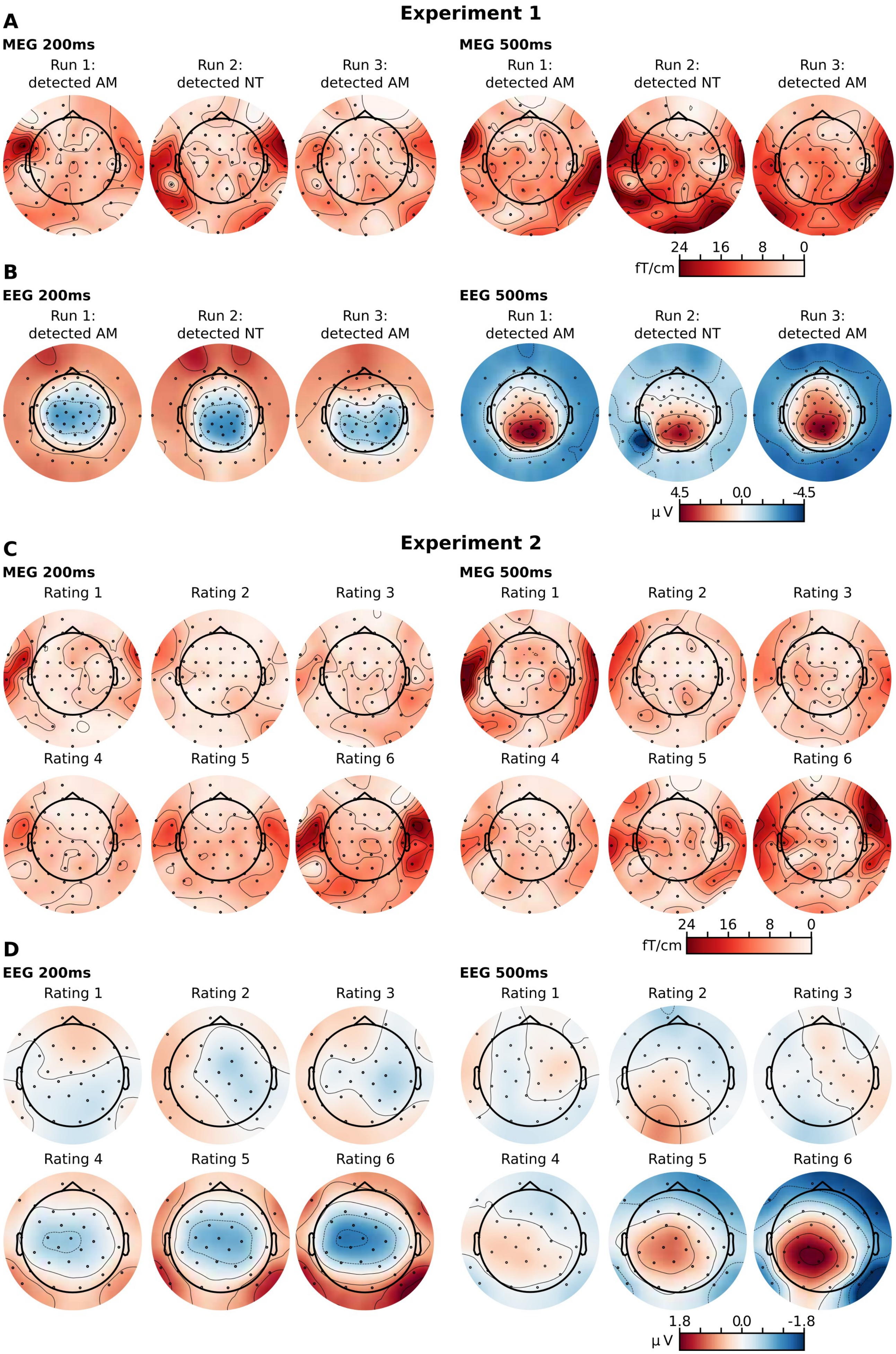
A
Experiment 1
MEG 200ms
MEG 500ms
Run 1: detected AM
Run 2: detected NT
Run 3: detected AM
Run 1: detected AM
Run 2: detected NT
Run 3: detected AM
24 16 8 0
fT/cm
B
EEG 200ms
EEG 500ms
Run 1: detected AM
Run 2: detected NT
Run 3: detected AM
Run 1: detected AM
Run 2: detected NT
Run 3: detected AM
4.5 0.0 -4.5
μV
C
Experiment 2
MEG 200ms
MEG 500ms
Rating 1
Rating 2
Rating 3
Rating 4
Rating 5
Rating 6
24 16 8 0
fT/cm
D
EEG 200ms
EEG 500ms
Rating 1
Rating 2
Rating 3
Rating 4
Rating 5
Rating 6
1.8 0.0 -1.8
μV

Figure S1. M/EEG topographies, related to Figure 2. Grand average topographies of the evoked potentials for MEG and EEG for both experiments. The first three columns show maps at 200ms, the last columns at 500ms after stimulus onset. For Experiment 1 (selective attention task), MEG (A) and EEG (B) maps are shown only for task-relevant, detected stimuli. For Experiment 2 (rating task, panels C and D), maps are shown for all target-present trials, sorted by rating.

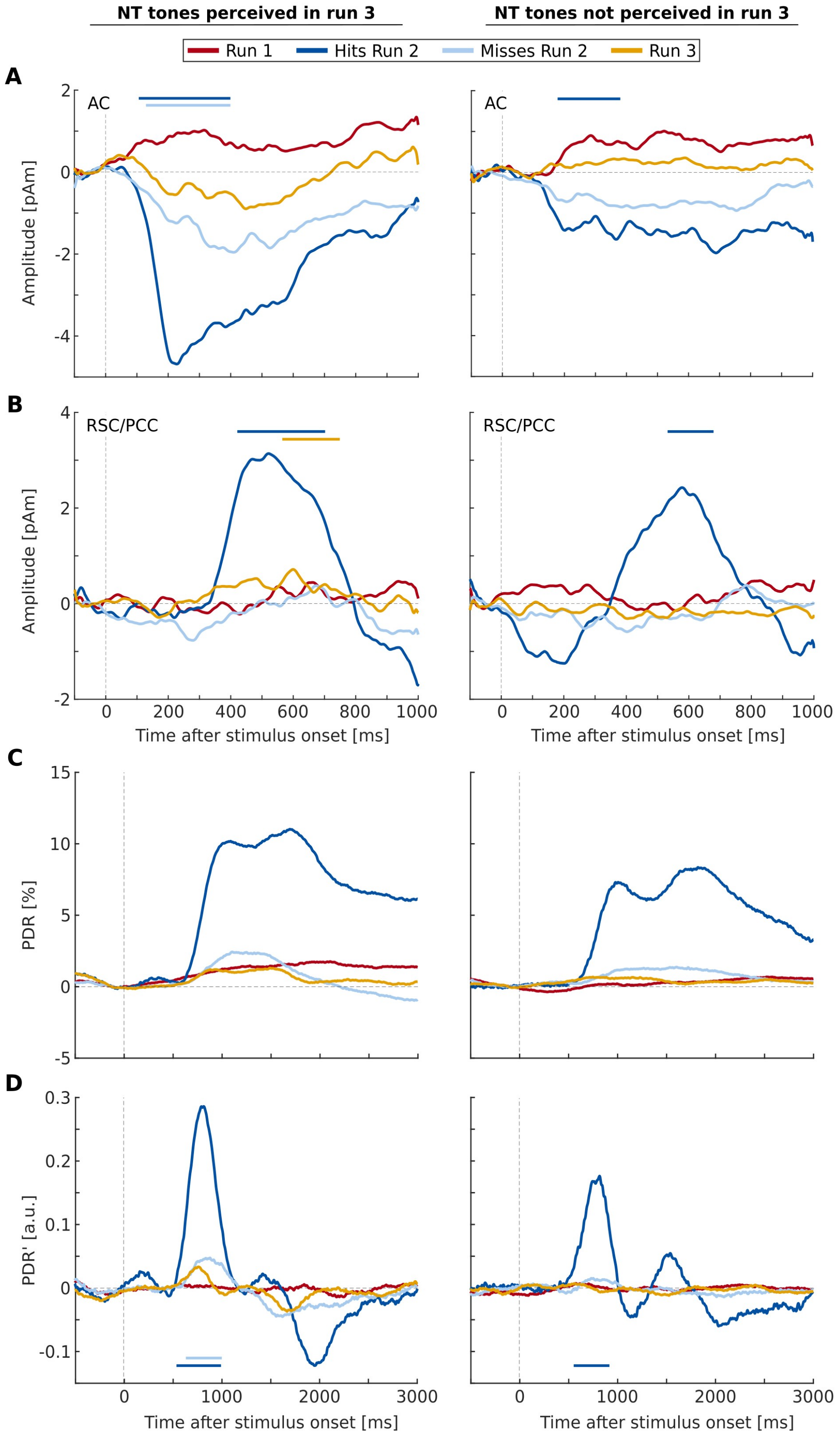

Figure S2. M/EEG and Pupil waveforms for NT tones in Experiment 1, related to Figure 3. The waveforms are averaged separately for the participants who perceived tones in the last run (left column, N=7) and those who did not (right column, N=7). Time courses for the NT tones in all runs are combined in one graph each for the AC (A), RSC/PCC (B), PDR (C), and first derivative of PDR (D). The colored horizontal bars in the waveform plots mark the time windows of significant activity in the time window of interest (permutation cluster test; AC: 100-400 ms, RSC/PCC: 300-800 ms, PDR': 500-1000 ms, no test for PDR).

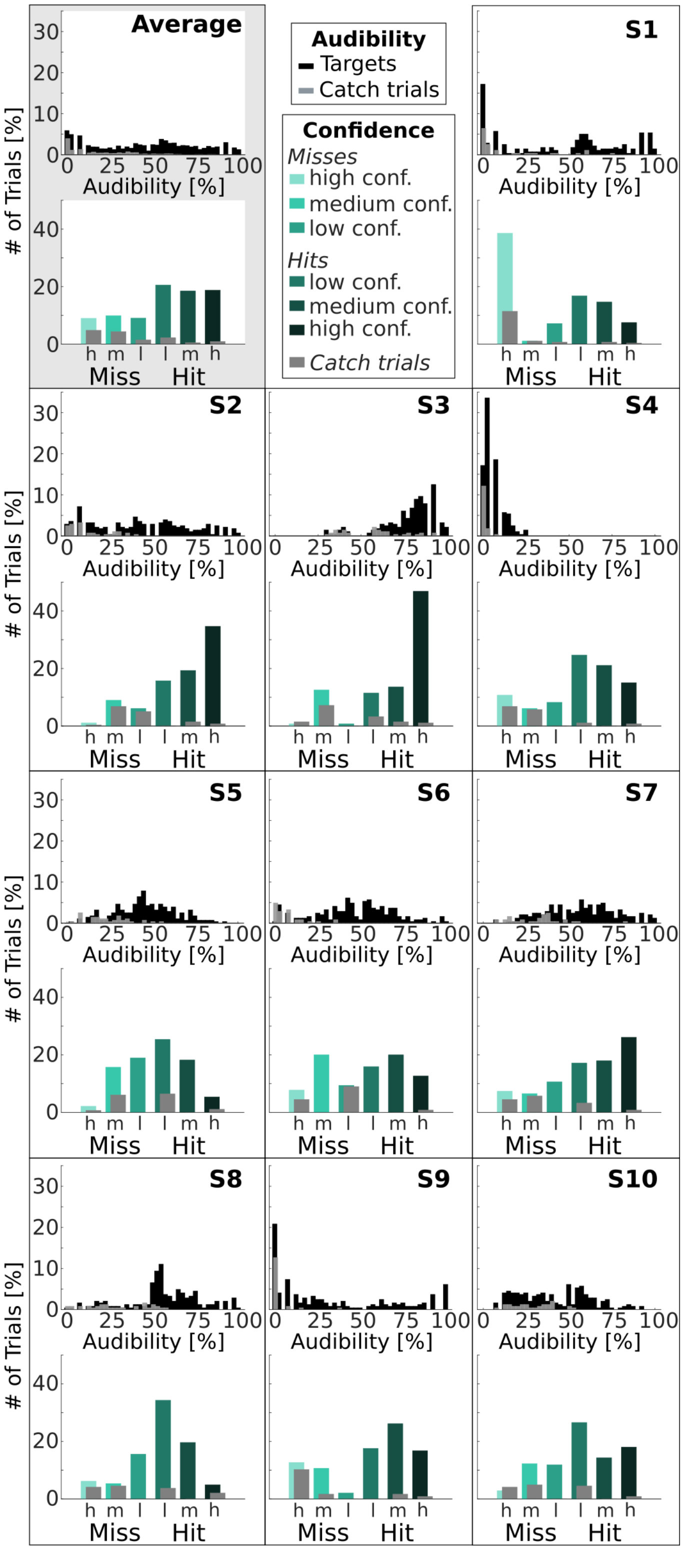

Figure S3. Rating scale comparison, related to Figure 5. In an additional psychoacoustic experiment, ten participants performed two runs of tone-in-noise detection. The paradigm as in Experiment 2 was used, only the response scales differed: In the first run, participants were asked to rate the audibility of the presented tone on a scale from 0 to 100%. In the second run, they used the same confidence rating scale as in Experiment 2. In both runs, 240 targets and 40 signal-absent trials were presented to the first five participants. For the remaining five participants, the number was reduced to 210 and 35 to reduce the total duration of the experiment. In each panel, the results of the audibility rating are shown in the upper graph, while the lower graph shows the confidence ratings. The upper left panel shows the group averages (N=10) for both ratings, the remaining panels are single participants’ data.

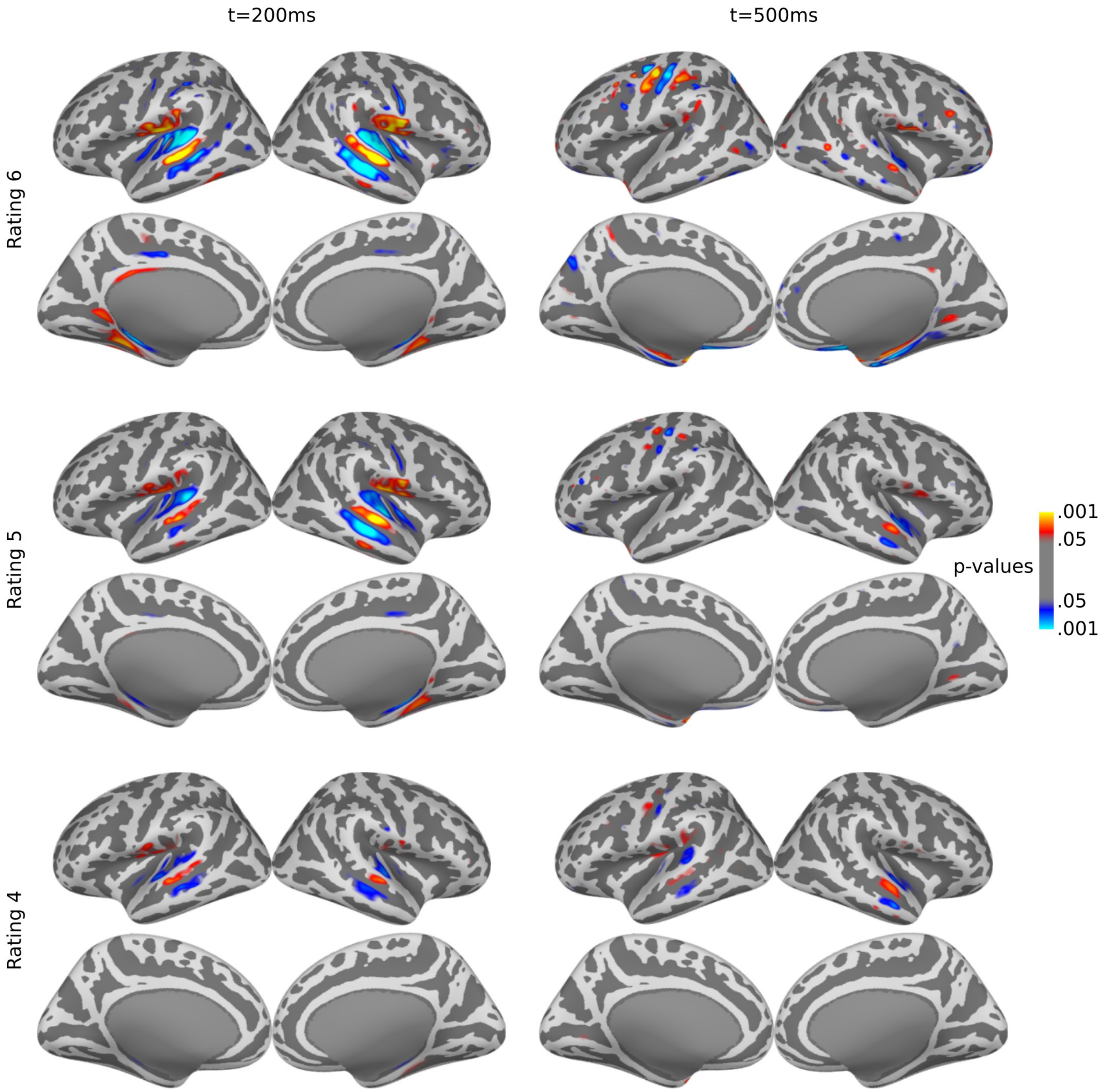

Figure S4. dSPM maps of Experiment 2, related to Figure 6**.** Maps are only shown for rating 4-6, because there was no significant activation for the other conditions. Compared to Experiment 1, the patterns are similar, but the amplitude is greatly reduced, especially in the RSC/PCC region. Likely reasons are the trial based structure with preceding noise onset and delayed button press, and the lower number of EEG channels, since the P3b is much more prominent in EEG than MEG.

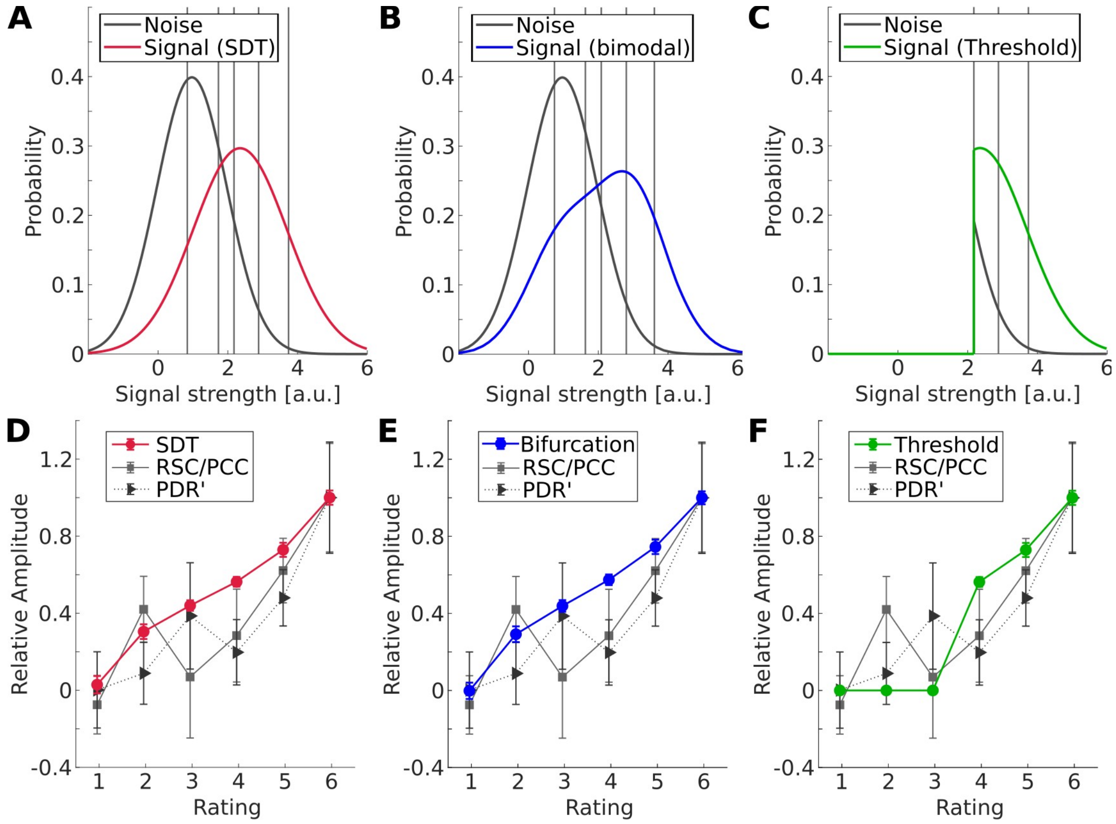

Figure S5. Modeling of RSC/PCC and PDR' amplitudes based on behavioral results of Experiment 2 (Rating task), related to Figure 7. (A) Signal (red) and noise (black) distribution fitted with a STD-Model based on the behavioral results. Vertical lines represent the group average of the five criteria calculated based on the two distributions. (D) Prediction of relative amplitudes (solid line/circles) and actual amplitudes of RSC/PCC activity (solid gray line and squares) and PDR' (dashed black line and triangles). All data points are averages across participants, error bars represent the standard error of the mean (N=17). The explained variance by the model is 0.67 and 0.63 for RSC/PCC and PDR', respectively. The inset shows predicted vs. measured amplitude for RSC/PCC (gray squares) and PDR' (black triangles). (B) and (E) are the equivalent plots for a model with a bimodal signal distribution[1]. (explained variance: 0.66 for RSC/PCC, and 0.46 for PDR'), (C) and (F) for a threshold model, which assumes no AC activity below threshold (explained variance: 0.66 for RSC/PCC, 0.61 for PDR'.

References

1. Sergent, C., Corazzol, M., Labouret, G., Stockart, F., Wexler, M., King, J.R., Meyniel, F., and Pressnitzer, D. (2021). Bifurcation in brain dynamics reveals a signature of conscious processing independent of report. Nat. Commun. *12, 1149*.
